# Biomarkers of lung epithelial injury and inflammation distinguish severe sepsis patients with acute respiratory distress syndrome

**DOI:** 10.1186/cc13080

**Published:** 2013-10-24

**Authors:** Lorraine B Ware, Tatsuki Koyama, Zhiguo Zhao, David R Janz, Nancy Wickersham, Gordon R Bernard, Addison K May, Carolyn S Calfee, Michael A Matthay

**Affiliations:** 1 Division of Allergy, Pulmonary and Critical Care Medicine, Department of Medicine, Vanderbilt University School of Medicine, T1218 MCN, 1161 21st Avenue S, Nashville, TN 37232-2650, USA; 2 Department of Pathology, Microbiology and Immunology, Vanderbilt University School of Medicine, Nashville, TN USA; 3 Department of Biostatistics, Vanderbilt University School of Medicine, Nashville, TN USA; 4 Division of Trauma and Surgical Critical Care, Department of Surgery, Vanderbilt University School of Medicine, Nashville, TN USA; 5 Cardiovascular Research Institute and Departments of Medicine and Anesthesia, University of California San Francisco, San Francisco, CA USA

## Abstract

**Introduction:**

Despite recent modifications, the clinical definition of the acute respiratory distress syndrome (ARDS) remains non-specific, leading to under-diagnosis and under-treatment. This study was designed to test the hypothesis that a biomarker panel would be useful for biologic confirmation of the clinical diagnosis of ARDS in patients at risk of developing ARDS due to severe sepsis.

**Methods:**

This was a retrospective case control study of 100 patients with severe sepsis and no evidence of ARDS compared to 100 patients with severe sepsis and evidence of ARDS on at least two of their first four ICU days. A panel that included 11 biomarkers of inflammation, fibroblast activation, proteolytic injury, endothelial injury, and lung epithelial injury was measured in plasma from the morning of ICU day two. A backward elimination model building strategy on 1,000 bootstrapped data was used to select the best performing biomarkers for further consideration in a logistic regression model for diagnosis of ARDS.

**Results:**

Using the five best-performing biomarkers (surfactant protein-D (SP-D), receptor for advanced glycation end-products (RAGE), interleukin-8 (IL-8), club cell secretory protein (CC-16), and interleukin-6 (IL-6)) the area under the receiver operator characteristic curve (AUC) was 0.75 (95% CI: 0.7 to 0.84) for the diagnosis of ARDS. The AUC improved to 0.82 (95% CI: 0.77 to 0.90) for diagnosis of severe ARDS, defined as ARDS present on all four of the first four ICU days.

**Conclusions:**

Abnormal levels of five plasma biomarkers including three biomarkers generated by lung epithelium (SP-D, RAGE, CC-16) provided excellent discrimination for diagnosis of ARDS in patients with severe sepsis. Altered levels of plasma biomarkers may be useful biologic confirmation of the diagnosis of ARDS in patients with sepsis, and also potentially for selecting patients for clinical trials that are designed to reduce lung epithelial injury.

## Introduction

The Acute Respiratory Distress Syndrome (ARDS) is a common clinical syndrome of acute lung inflammation, non-cardiogenic pulmonary edema and acute respiratory failure [1]. Despite recent modifications [2] to the American European Consensus Conference (AECC) definition [3], the clinical definition of ARDS remains non-specific and is not uniformly applied. As a result, ARDS remains underdiagnosed and undertreated.

The discovery and validation of biomarkers of myocardial injury and ventricular overload such as troponin and brain-natriuretic peptide (BNP) has transformed the diagnosis, management and design of clinical trials in conditions such as myocardial infarction and congestive heart failure. In a similar way, identification of plasma biomarkers that facilitate diagnosis of ARDS could improve clinical care, enhance our understanding of pathophysiology, and could be used to enroll a more homogeneous group of patients into clinical trials of new therapies, increasing the likelihood of detecting a treatment effect. Although several plasma biomarkers have been studied in ARDS [4], the majority of studies have focused on prognosis, rather than diagnosis. In addition, given the complex pathophysiology of ARDS [5], it is unlikely that a single biomarker will have adequate specificity for ARDS. Indeed, several recent studies in ARDS have shown the superiority of a multiple biomarker approach for diagnosis in patients with trauma [6] and for prognosis in established ARDS due to a variety of causes [7,8]. Several plasma biomarkers have been studied in patients with ARDS, but no studies have tested the possible value of a panel of plasma biomarkers in patients with severe sepsis who have developed ARDS by clinical criteria and determined if a combination of abnormal biomarkers could be used for confirming the diagnosis of ARDS on biologic grounds.

The current study was designed to test the hypothesis that a biomarker panel would be useful for biologic confirmation of the clinical diagnosis of ARDS in patients at risk of developing ARDS due to severe sepsis. We also determined whether biomarkers that performed well for diagnosis of ARDS in patients with severe trauma have value in severe sepsis, an important consideration since biomarker levels have been shown to differ substantially between traumatic and non-traumatic ARDS [9].

## Materials and methods

### Study design and patient selection

This study is a retrospective nested case control study within the Validating Acute Lung Injury bIomarkers for Diagnosis (VALID) study. VALID is a 2,500 patient prospective cohort study that has been enrolling critically ill patients in the Vanderbilt Medical, Surgical, Trauma and Cardiovascular ICUs since 2006 [10-12]. Patients are enrolled on the morning of ICU day 2 if they are not being transferred out of the ICU. At the time of enrollment, plasma is obtained for biomarker measurement. Comprehensive clinical data are collected for the first four ICU days including severity of illness scoring (Simplified Acute Physiology Score II (SAPS II) [13], Acute Physiology and Chronic Health Evaluation II (APACHE II) [14]), daily laboratory values, hemodynamics, ventilator settings, medications and daily phenotyping for severe sepsis, ARDS and other organ failures. Thereafter, comprehensive clinical outcomes are collected including duration of mechanical ventilation, length of ICU and hospital stay, hospital mortality and long term mortality. The VALID study is approved by the Vanderbilt Institutional Review Board. Informed consent is obtained from patients or their surrogates; if patients are unable to consent and no surrogates can be identified then the Institutional Review Board has granted a waiver of informed consent for this minimal risk study.

For the current case control study, 100 patients with severe sepsis and no evidence of ARDS (defined as not meeting AECC acute lung injury (ALI) or ARDS criteria) during the first four ICU days serve as controls and 100 patients with severe sepsis and evidence of ARDS (defined as meeting AECC ALI or ARDS criteria) on at least two of the first four ICU days serve as cases. For some analyses, cases were further restricted to patients who had ARDS at the time of blood draw on the morning of ICU day 2 (n = 91 pairs) or to the most severely ill patients who met ARDS criteria on all four days (n = 66 pairs). To minimize confounding of biomarker associations with ARDS by clinical variables, such as severity of illness, cases and controls were one-to-one matched for severity of illness (APACHE II, within one point), age (within 10 years), gender (one-to-one match) and number of non-pulmonary organ failures (within one).

### Definitions of severe sepsis and ARDS

For inclusion as a case or control, patients were required to have severe sepsis at enrollment as defined by consensus definitions [15]. The presence or absence of ARDS was determined daily by review of all chest radiographs and blood gases in the past 24 hours using criteria set forth by the AECC [3]. When no blood gases were available the SpO_2_/FiO_2_ ratio was utilized [16]. All chest radiographs were reviewed by consensus of two trained physician investigators. When patients met chest radiograph and oxygenation criteria for ARDS, then the medical record was thoroughly reviewed for any evidence of a primary cardiogenic cause of pulmonary edema [17]; patients with cardiogenic pulmonary edema were excluded.

### Biomarker selection and assays

Because there are very few prior studies of biomarkers for diagnosis of ARDS, we selected a panel of biomarkers that included top performing biomarkers from our recent study in trauma-associated ARDS [6] (receptor for advanced glycation end products (RAGE), procollagen peptide III (PCPIII), BNP, angiopoietin-2 (ANG2) and IL-8) as well as other biomarkers of lung epithelial injury and inflammation (surfactant protein D (SPD), club cell secretory protein (CC16, formerly known as Clara cell secretory protein) and IL-6), and biomarkers that have been associated with other lung diseases (matrix metalloproteases (MMP)-1, -3 and -9) [18-20]. All biomarkers were assayed in duplicate in thawed plasma that was collected at VALID enrollment using commercially available singleplex ELISAs (RAGE and ANG2, R&D Systems Minneapolis, MN, USA; SP-D, Yamasa Corporation, Tokyo, Japan; CC16, BioVendor, Candler, NC, USA; BNP, Peninsula Laboratories, San Carlos, CA, USA), multiplex ELISAs (IL-6, IL-8, MMP-1, MMP-3, MMP-9, Meso Scale Discovery, Rockville, MD, USA) or radioimmunoassay (PCPIII, Peninsula Laboratories, San Carlos, CA, USA).

### Statistical analysis

Demographic and clinical variables were assessed using Wilcoxon signed rank tests for continuous variables and McNemar’s tests for categorical variables accounting for pairing of the cases and controls. Biomarker values underwent logarithmic transformation to reduce right skewness. Values below the detection limit were imputed at half the lower limit of detection for each biomarker. We used a backward elimination model-building strategy on 1,000 bootstrapped data to select the biomarkers for further consideration in the logistic regression model. For each bootstrap sample, a full model with all 11 variables in consideration was fit. Then, the variable with the largest *P* value (Wald test) was dropped, and a new model was fit with one fewer variables. This backward elimination process was repeated until only one variable was retained. The predictors were then ranked from most significant (the last one to remain) to least significant (the first one eliminated). The average rank from 1,000 bootstrap repetitions was used to select the variables for further consideration. Using the best five variables selected in the previous steps, a multivariable conditional logistic model was fit. The final model included all variables selected in the previous steps; no reduction of the model was attempted at this stage. From each model, we computed the predicted probability of ARDS for each individual and computed receiver operating characteristics (ROC) curves and their area under the curve (AUC). We validated each model using a bootstrap method (10,000-iteration) and reported the bootstrap bias-corrected AUCs with 95% bootstrap confidence interval (CI) (15). All analyses were performed with R version 2.13.0 (R Foundation for Statistical Computing, Vienna, Austria).

## Results

### Patient characteristics

Patient demographic and clinical characteristics are summarized in Table 1. ARDS cases and sepsis controls were well matched (by study design) for age, gender, APACHE II score and number of non-pulmonary organ failures at enrollment. Compared to sepsis controls, ARDS cases were more likely to be Caucasian, had fewer days alive and free of mechanical ventilation (ventilator-free days) and had trends towards longer ICU stays and higher hospital mortality (Table 1). Among the ARDS cases, 91 had onset on ICU day 1 and the remainder had onset on ICU day 2. Severity of ARDS as assessed by the AECC criteria was high, with the majority (79%) of cases meeting ARDS criteria (PaO_2_/FiO_2_ ≤200) on the day of onset and the minority (21%) meeting only ALI criteria (200 < PaO_2_/FiO_2_ ≤300) on the day of onset.

**Table 1:** Demographic and clinical characteristics of 100 severe sepsis patients with ARDS (cases) and 100 severe sepsis patients without ARDS (controls)

**Characteristic**	**ARDS casesnumber = 100**	**Controlsnumber = 100**	***P*** **Value**
Age	56 (51, 65)	59 (51, 65)	0.97
Male	52 (52%)	52 (52%)	1.0
Caucasian	89 (89%)	79 (79%)	0.10
Current smoker	35 (35%)	40 (40%)	0.56
APACHE II	28 (24, 32)	28 (24, 32)	1.0
Source of ICU admit			
Emergency room	27 (27%)	37 (37%)	0.027
Hospital floor	40 (40%)	21 (21%)	
Other hospital	23 (23%)	22 (22%)	
Operating room	9 (9%)	17 (17%)	
Other	1 (1%)	3 (3%)	
Day of ALI onset			
ICU day 1	91 (91%)	NA	
ICU day 2	9 (9%)		
Mechanical ventilation^a^	83 (83%)	58 (58%)	<0.001
Any ventilatory support^b^	89 (89%)	59 (59%)	<0.001
Non-pulmonary organ failure at enrollment	92 (92%)	91 (91%)	1.0
Vasopressors at enrollment	44 (44%)	43 (43%)	0.50
ICU stay (days)	9 (5, 13)	5 (3, 11)	0.003
Ventilator-free days	18 (2, 24)	25 (7, 28)	<0.001
Hospital mortality	29 (29%)	27 (27%)	0.87

Data as median (1^st^ and 3^rd^ quartiles) or Number (%) as indicated. NA = not applicable. ^a^Mechanical ventilation on any study day; ^b^mechanical ventilation or noninvasive ventilation on any study day. ALI, acute lung injury; APACHE II, Acute Physiology and Chronic Health Evaluation II; ARDS, acute respiratory distress syndrome.

### Biomarker values

**Figure 1:**
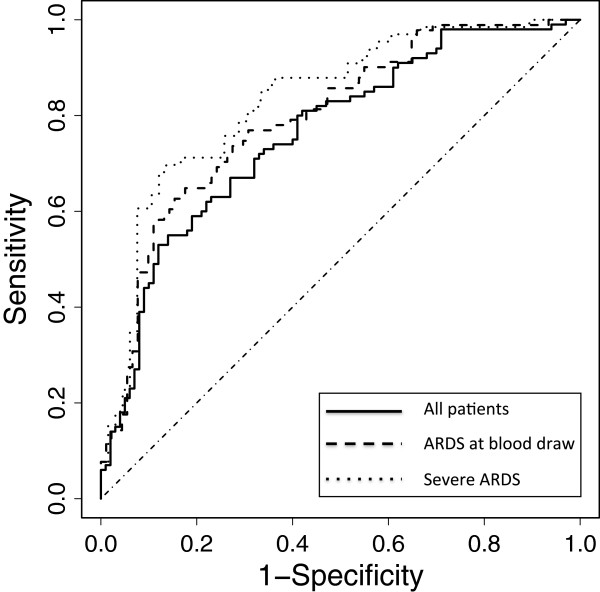
**Receiver operator characteristic (ROC) curve analysis of the plasma biomarker panels for differentiating ARDS (cases) from controls.** Predicted probability of ARDS for each subject was computed from a logistic regression model that includes the top five biomarkers (SP-D, RAGE, IL-8, CC-16 and IL-6). Specificity and sensitivity were computed at each possible cutoff of the predicted probability. Three ROC analyses are shown. The solid line shows the ROC analysis for all 200 patients in the study (100 cases, 100 controls). The AUC is 0.75 (95% CI: 0.7 to 0.84). The dashed line shows the ROC analysis using only the 91 cases who had ARDS at the time of the blood draw for biomarker measurement as well as their matched controls. The AUC for this model is 0.78 (95% CI: 0.74 to 0.87). The dotted line shows the ROC analysis using only the 66 patients who had the most severe ARDS (ARDS on all study days) and their matched controls. The AUC for this model is 0.82 (95% CI: 0.77 to 0.90). ROC, receiver operator characteristic curve; ARDS, acute respiratory distress syndrome; SP-D, surfactant protein D; RAGE, receptor for advanced glycation endproducts; IL-8, interleukin 8; CC16, club cell protein-16; IL-6, interleukin 6; AUC, area under the receiver operator characteristic curve; CI, confidence interval.

**Figure 2:**
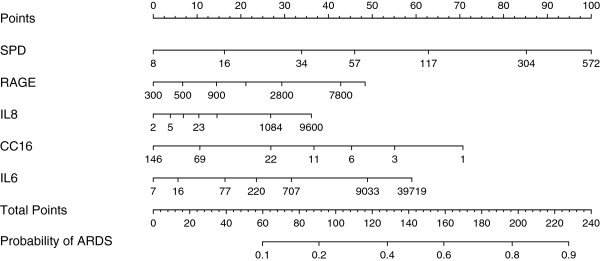
**The multivariable logistic regression model was used to create a prediction model nomogram for the probability of ARDS.** A value in each biomarker predictor variable corresponds to a point scale at the top. The sum of the individual predictor variable points corresponds to the total points and the probability of ARDS shown at the bottom. For each predictor variable, the shown values are approximately 1st, 5th, 25th, 50th, 75th, 95th, and 99th percentiles. ARDS, acute respiratory distress syndrome.

Comparison of biomarker values between cases and controls is summarized in Table 2. Five biomarkers were significantly different (*P* <0.05) in univariable analysis between cases and controls including SP-D, RAGE, IL-8, CC-16 and IL-6. These five biomarker variables were selected in the model building step and used to construct a diagnostic model for ALI (Table 3). Model performance was evaluated by ROC curve analysis. Using the top five biomarkers (SP-D, RAGE, IL-8, CC-16 and IL-6), the AUC was 0.75 (95% CI: 0.7 to 0.84) (Table 3, Figure 1) for the diagnosis of ARDS. By contrast, the AUC for diagnosis using single biomarkers was poor with AUCs ranging from 0.59 for IL-6 to 0.69 for SP-D (Table 3). A nomogram that illustrates the potential clinical use of the five-biomarker panel is illustrated in Figure 2.

**Table 2:** Comparison of plasma biomarker levels between 100 severe sepsis patients with ARDS (cases) and 100 severe sepsis patients without ARDS (controls)

**Biomarker**	**Number**	**ARDS cases number = 100**	**Controls number = 100**	***P*** **Value**
SP-D (ng/ml)	200	86 (46 to 159)	43 (28 to 77)	<0.001
RAGE (pg/ml)	200	1,844 (1,060 to 3,737)	1,232 (766 to 2147)	<0.001
IL-8 (pg/ml)	200	27.2 (14.2 to 119.5)	16.8 (9.3 to 45.6)	0.006
CC16 (ng/ml)	200	9.2 (5.0 to 15.8)	13.1 (6.3 to 27.8)	0.013
IL-6 (pg/ml)	200	283 (92 to 803)	142 (57 to 550)	0.023
PCPIII (ug/ml)	200	11.5 (6.9 to 18.7)	11.5 (6.7 to 34.2)	0.25
BNP (pg/ml)	200	404 (264 to 748)	412 (212 to 712)	0.38
MMP-9 (ng/ml)	195	151 (67 to 300)	163 (81 to 337)	0.39
MMP-1 (ng/ml)	195	28 (15 to 51)	31 (17 to 56)	0.44
Ang2 (ng/ml)	200	13 (7 to 21)	12 (7 to 20)	0.67
MMP-3 (ng/ml)	195	22 (15 to 35)	22 (14 to43)	0.78

Data as median (interquartile range). ARDS, acute respiratory distress syndrome; SP-D, surfactant protein D; RAGE, receptor for advanced glycation endproducts; IL-8, interleukin 8; CC16, club cell protein-16; IL-6, interleukin 6; PCPIII, procollagen peptide III; BNP, brain natriuretic peptide; MMP-9, matrix metalloprotease 9; MMP-1, matrix metalloprotease 1; Ang2, angiopoietin 2; MMP-3, matrix metalloprotease 3.

**Table 3:** Comparison of models for diagnosis of ARDS using single biomarkers to a combined model utilizing the top five performing biomarkers

**Model**	**All data (100 pairs)**	**Enrollment day cases (91 pairs)**	**Severe cases only (66 pairs)**
	**AUC (95% CI)**^**a**^	**AUC (95% CI)**^**a**^	**AUC (95% CI)**^**a**^
Single marker models			
SPD	0.69 (0.6, 0.76)	0.71 (0.63, 0.79)	0.72 (0.62, 0.81)
RAGE	0.64 (0.56, 0.72)	0.68 (0.6, 0.75)	0.67 (0.57, 0.76)
IL8	0.61 (0.54, 0.69)	0.63 (0.55, 0.7)	0.64 (0.55, 0.73)
CC16	0.60 (0.52, 0.68)	0.60 (0.52, 0.68)	0.64 (0.55, 0.74)
IL6	0.59 (0.52, 0.67)	0.61 (0.53, 0.69)	0.63 (0.53, 0.72)
Multivariable model (includes SPD, RAGE, IL-8, CC16, IL6)	0.75 (0.7, 0.84)	0.78 (0.74, 0.87)	0.82 (0.77, 0.9)

^a^All AUCs are bootstrap bias-corrected; all 95% CI are bootstrap CIs. AUC, area under the receiver operator characteristic curve; CI, confidence interval; ARDS, acute respiratory distress syndrome; SP-D, surfactant protein D; RAGE, receptor for advanced glycation endproducts; IL-8, interleukin 8; CC16, club cell protein-16; IL-6, interleukin 6.

Nine of the patients included as cases had onset of ARDS after plasma was obtained. To determine if the biomarker panel performed better if plasma was obtained when ARDS was already established, we repeated the analysis excluding the nine cases with onset of ARDS on ICU day 2 as well as their matched controls. In this analysis, the model with the top five biomarkers (SP-D, RAGE, IL-8, CC-16 and IL-6) had an AUC of 0.78 (95% CI: 0.74 to 0.87) (Table 3, Figure 1). As a sensitivity analysis, we repeated the analysis using only the most severe cases (n = 66) who met ARDS criteria on all four days in the ICU and their matched controls. In this analysis the model with the top five biomarkers (SP-D, RAGE, IL-8, CC-16 and IL-6) had an AUC of 0.82 (95% CI: 0.77 to 0.90) (Table 3, Figure 1) compared to single biomarker performance AUCs ranging from 0.63 (IL-6) to 0.72 (SP-D). We also assessed the sensitivity and specificity of the diagnostic models. Setting the sensitivity at 70%, the specificity of the diagnostic model that included all patients was 68%. Specificity improved to 75% and 83% when the analysis was restricted to patients with ARDS on enrollment day or to patients with the ARDS on all four study days, respectively.

## Discussion

The diagnosis of ARDS is based on clinical definitions that lack both sensitivity and specificity. The goal of the current study was to test the performance of a panel of biomarkers for the diagnosis of ARDS and to test if these plasma markers would provide biologic confirmation of the clinical diagnosis. To reduce clinical heterogeneity, we focused on patients with severe sepsis, the most common and most lethal underlying etiology of ARDS [21]. As hypothesized, a logistic regression model that utilized a panel of biomarkers had substantially superior performance to single biomarkers for differentiating sepsis patients with ARDS from those without ARDS as evaluated by ROC curve analysis. From among the 11 biomarkers tested in this exploratory study, a panel that included the five top-performing biomarkers (SP-D, RAGE, IL-8, CC-16 and IL-6) had an AUC of 0.75 (95% CI: 0.7 to 0.84) for the diagnosis of ARDS. Performance of the biomarker panel was further enhanced when only patients with ARDS at the time of blood draw (AUC 0.78) or patients with the most severe ARDS (AUC 0.82) were considered.

The best performing biomarkers for the diagnosis of ARDS in the current study of patients with severe sepsis were different from the best performing biomarkers identified in a similar study in patients with severe trauma [6], although there was some overlap. In the trauma study, the best performing biomarkers were RAGE, PCPIII, BNP, ANG2, IL10, TNF-α, and IL8 with an AUC of 0.86 for differentiating patients with ARDS from critically ill trauma patients without ARDS. Two biomarkers, RAGE and IL-8, contributed to diagnostic models in both studies. In trauma patients, biomarkers of fibroblast activation (PCPIII), endothelial injury (ANG2), other inflammatory markers (IL-10, TNF) and heart failure (BNP, lower in ARDS) were also useful for differentiating cases from controls whereas in sepsis, biomarkers of lung epithelial injury (SPD, RAGE, CC16) and inflammation (IL6, IL8) predominate. These differences may reflect important differences in the pathophysiology of both the underlying conditions (trauma and sepsis) as well as differences in the pathophysiology of ARDS in these different clinical settings.

Three of the top five performing biomarkers in the current study were biomarkers of lung epithelial injury. SP-D is a normal constituent of surfactant that is produced almost exclusively by the alveolar epithelial type II cell. Elevated levels of SP-D have been reported in the circulation in patients with ARDS compared to those with hydrostatic pulmonary edema [22] and higher levels have been independently associated with 180-day mortality and reduced ventilator and organ-failure free days in patients with ARDS [23]. RAGE, although ubiquitously expressed, is most highly expressed by the type I alveolar cell [24]. Similar to SP-D, higher plasma levels of RAGE have been associated with adverse outcomes in patients with ARDS [25]. CC16 is a small 16 kDa protein secreted by the club cells of the distal airway (previously known as Clara cells). In contrast to other lung epithelial markers, lower levels of CC16 have previously been associated with ARDS [26] although one small study reported an increase in levels at the time of onset of ARDS in patients with ventilator-associated pneumonia [27]. In the current study, lower levels of CC16 were associated with the diagnosis of ARDS. Taken together with prior studies of lung epithelial markers and prognosis of ARDS, the current findings indicate that alterations in the plasma levels of biomarkers of lung epithelial injury are key features that can be used to differentiate both the presence and the severity of ARDS in patients with sepsis. Measures of lung epithelial injury might be particularly useful in selecting patients likely to benefit from lung-epithelial targeted therapies such as keratinocyte growth factor [28-30] in future clinical trials in ARDS.

This study has both strengths and limitations. Major strengths include the detailed, daily prospective patient phenotyping for ARDS as part of the VALID cohort study, and the matching of cases and controls for important clinical characteristics, such as age, gender, severity of illness and number of non-pulmonary organ failures. This stringent matching of cases and controls eliminates severity of illness as the primary determinant of differences in biomarker levels in this exploratory study, thus making it more likely that biomarkers that truly reflect the presence of ARDS have been identified. Limitations of the study include the retrospective single center design and the case control study design. A case control design is more likely to overestimate the association between a given biomarker and the diagnosis of ARDS compared to a prospective cohort design. In addition, the study included only 11 biomarkers and thus was not an exhaustive examination of all potential biomarkers that might be useful for the diagnosis of ARDS. Nevertheless, the simultaneous comparison of performance of 11 biomarkers for the diagnosis of ARDS in patients with severe sepsis provides important new information about the relative performance of a variety of plasma biomarkers of different aspects of the pathophysiology of ARDS, information that has not previously been available from the many single biomarker studies in ARDS.

## Conclusions

In conclusion, abnormal levels of five biomarkers in plasma provided excellent discrimination for the diagnosis of ARDS in patients with severe sepsis as assessed by ROC curve analysis. Three of the five biomarkers were generated by the lung epithelium, suggesting that lung epithelial injury is a critical determinant of alveolar flooding and the subsequent arterial hypoxemia and bilateral opacities that constitute the clinical definition of ARDS, a finding that is concordant with evidence that impaired alveolar epithelial fluid clearance is characteristic of patients with ARDS [31,32]. Although the definition of ARDS is based on clinical criteria, altered levels of plasma biomarkers may be useful to assist in confirming the diagnosis in patients with shock and possible sepsis, and also potentially selecting patients for clinical trials that are designed to reduce lung epithelial injury [5,28]. The biomarker panel might also be useful to categorize patients with sepsis-induced ARDS by their biologic profile as well as their clinical profile, as has been done recently in other lung disease, such as asthma [33].

## Key messages

● Among a panel of 11 biomarkers of various aspects of the pathophysiology of ARDS, biomarkers of lung epithelial injury and inflammation were the most useful for discriminating sepsis patients with ARDS from those without ARDS.

● A five biomarker panel that included SP-D, RAGE, CC-16, IL-8 and IL-6 had an area under the ROC curve of 0.75 (95% CI: 0.7 to 0.84) for diagnosis of ARDS.

● For diagnosis of more severe ARDS the area under the ROC curve was 0.82 (95% CI: 0.77 to 0.90).

● Altered levels of plasma biomarkers may be useful biologic confirmation of the diagnosis of ARDS in patients with sepsis.

● A biomarker panel that includes biomarkers of lung epithelial injury and inflammation may be useful for selecting patients for clinical trials that are designed to reduce lung epithelial injury.

## Abbreviations

AECC: American European consensus conference; ALI: Acute lung injury; ANG-2: Angiopoietin-2; APACHE II: Acute physiology and chronic health evaluation II; ARDS: Acute respiratory distress syndrome; AUC: Area under the receiver operator characteristic curve; BNP: Brain natriuretic peptide; CC16: Club cell secretory protein; ELISA: Enzyme- linked immunosorbent assay; IL-6: Interleukin 6; IL-8: Interleukin 8; MMP: Matrix metalloprotease; PCPIII: Procollagen peptide III; RAGE: Receptor for advanced glycation endproducts; ROC: Receiver operator characteristic; SAPS II: Simplified acute physiology score II; SP-D: Surfactant protein D; TNF-α: Tumor necrosis factor alpha; VALID: Validation of acute lung injury biomarkers for diagnosis study.

## Competing interests

The authors declare that they have no competing interests.

## Authors’ contributions

LBW designed the study, oversaw the data acquisition and analysis and drafted the manuscript. TK and ZZ designed the study and performed the data analysis. DR collected and interpreted patient data for the study. NW made the biomarker measurements. GB designed the study and assisted with interpretation of results. AM enrolled patients in the study and assisted with interpretation of results. CC and MM designed the study and assisted with interpretation of results. All authors read and approved the final manuscript.
